# Symmetry Analysis of Amputee Gait Based on Body Center of Mass Trajectory and Discrete Fourier Transform

**DOI:** 10.3390/s20082392

**Published:** 2020-04-23

**Authors:** Claudia Ochoa-Diaz, Antônio Padilha L. Bó

**Affiliations:** 1 Gama Engineering College, University of Brasilia, Brasilia 70910-900, Brazil; 2 LARA/Department of Electrical Engineering, University of Brasilia, Brasilia 70910-900, Brazil; antonio.plb@uq.edu.au; 3 School of Information Technology and Electrical Engineering, The University of Queensland, Brisbane 4072, Australia

**Keywords:** center of mass, lower-limb amputation, kinematics, Fourier analysis

## Abstract

The calculation of symmetry in amputee gait is a valuable tool to assess the functional aspects of lower limb prostheses and how it impacts the overall gait mechanics. This paper analyzes the vertical trajectory of the body center of mass (CoM) of a group formed by transfemoral amputees and non-amputees to quantitatively compare the symmetry level of this parameter for both cases. A decomposition of the vertical CoM into discrete Fourier series (DFS) components is performed for each subject’s CoM trajectory to identify the main components of each pattern. A DFS-based index is then calculated to quantify the CoM symmetry level. The obtained results show that the CoM displays different patterns along a gait cycle for each amputee, which differ from the sine-wave shape obtained in the non-amputee case. The CoM magnitude spectrum also reveals more coefficients for the amputee waveforms. The different CoM trajectories found in the studied subjects can be thought as the manifestation of developed compensatory mechanisms, which lead to gait asymmetries. The presence of odd components in the magnitude spectrum is related to the asymmetric behavior of the CoM trajectory, given the fact that this signal is an even function for a non-amputee gait. The DFS-based index reflects this fact due to the high value obtained for the non-amputee reference, in comparison to the low values for each amputee.

## 1. Introduction

Most of the people who have suffered from amputation and become users of lower limb prostheses tend to maximize the capacities of the contralateral limb to counteract the limitations of the prosthetic device. These compensatory strategies may result in serious consequences that could yield new physical impairments, like osteoarthritis on the joints of the intact limb [1,2], osteopenia and osteoporosis on the amputee limb [3], as well as low back pain [4]. The measurement of kinematic and kinetic variables have identified common compensatory strategies developed by users of lower limb prostheses. The most frequent strategies found, specifically in transfemoral amputees, are full extended knee on the prosthetic side, greater vertical ground reaction forces (GRF), increased hip angle values on the intact side, among others [5]. These strategies reflect an asymmetric condition that deviates the affected variables behavior from the normal patterns.

Asymmetries on amputees have been often investigated in terms of spatio-temporal variables [6,7], joint kinematics [8,9], joint kinetics [10,11], and GRF [6,7,12]. In these works, the assessment of the symmetry level is mostly calculated using the symmetry index, originally proposed by [13]. The index calculation takes one of the sides as a reference and simply calculates the difference between the values from the two sides and divide it by their average. Some variation of this index has been proposed by other authors [14,15]. The main disadvantage of these methods is that its outcome is strongly affected by the choice of the reference side [16]. Another types of methods are used when two waveforms need to be compared during a period of time. These techniques may include the use of correlation coefficients [17], principle components [18], or frequency domain analyses [19].

A less common studied variable that also exhibits the effects of compensatory mechanisms in amputee gait is the body Center of Mass (CoM). As an anatomical point that reflects the entire body mechanics, the trajectory of the CoM has an asymmetric behavior between consecutive steps when certain compensations take place. Some related works investigate how this parameter is affected by other gait variables under an amputee condition. In [20], the relationship between the external mechanical cost, the vertical excursion of the CoM and the knee angle in the sagittal plane was studied. The vertical CoM from amputees showed an amplitude four times greater than the normal values from able-bodied subjects, besides a different shape from the observed sine-wave shape in the non-amputee case. In this same line, a dynamic walking model was used in [21] to predict how weak push-off work could affect the CoM mechanics in unilateral transtibial amputees. The CoM measurement can also serve to additional calculations of gait performance, like the overall metabolic cost of walking, which provides an estimation of energy expenditure [22,23,24].

Despite the asymmetric condition of amputee gait, the trajectory of the CoM still preserves its periodicity between strides, which favors the use of the discrete Fourier series (DFS) to mathematically represent the performance of this parameter during a gait cycle. In this work, a spectral-based method, which is often employed to analyze oscillatory movements, such as tremor [25], is proposed. The method is used to examine the vertical CoM trajectory of a group formed by transfemoral amputees and non-amputees, while the latter are considered for method validation. Additionally, in order to quantify the symmetry level of the CoM trajectory of each studied subject, an index based on the DFS is calculated. The index value reflects what is exhibited on the different CoM waveforms. In contrast with other approaches, the proposed method does not require obtaining discrete gait features. Although such segmentation may be performed automatically [26], errors may be frequent when signals from daily activities are used.

In this work, we present a feasibility study in which a spectral-based method is used to investigate the CoM trajectory as a measure of gait symmetry in amputee gait. Our hypothesis is that such method will be able to assess asymmetric patterns and later be employed to monitor interventions targeting the reduction of secondary complications after transfemoral amputations.

## 2. Materials and Methods

### 2.1. Subjects

The participants of this study are divided into two groups of amputees and non-amputees. The amputee group was formed by three male subjects. The inclusion criterion was unilateral transfemoral amputation caused by trauma or malignancy. All amputees also used the same knee and foot prosthesis model for the last five years: 3R80 knee joint and 1C30 Trias Foot (Otto Bock, Germany). The control group was constituted by five male subjects with the same fitness level indicated by three weekly sessions of aerobic exercise on average and no previous or current diagnosed movement disorder, joint pain, or injury.

The mean height (±SD) is 1.75 (±0.02 m) and 1.78 (±0.06 m) for the non-amputee and the amputee volunteers, respectively. The mean body mass is 74.8 (±8.3 kg) for the non-amputees and 78.17 (±5.48 kg) for the amputee group. The mean age is 29.38 (±2.26 years) for all subjects. Volunteers were informed of all purposes, procedures, benefits, and risks of the study and signed an informed consent form. The research was approved by the Ethics Committee of the Faculty of Health Science at the University of Brasilia, in accordance with the Helsinki Declaration.

### 2.2. Experimental Protocol and Data Analysis

**Figure 1:**
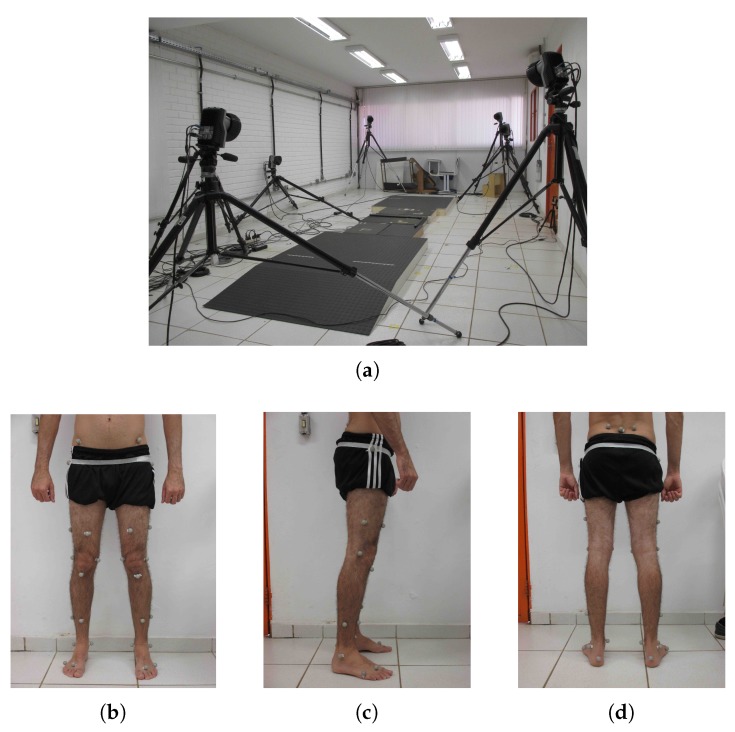
The experimental setup: (**a**) the walkway with three force platforms and eight cameras of the motion capture system around it. The marker set used on the subjects: (**b**) frontal, (**c**) lateral, and (**d**) posterior view.

The participants of this study were asked to walk at their normal walking speed over a walkway with a length of 3 m, where three instrumented force platforms (Bertec, Columbus, OH, USA) were embedded along the path to collect the GRF information of each step (see Figure 1a). At the same time, a motion capture (MOCAP) system (Qualisys, Inc., Göteborg, Sweden) was responsible for recording the trajectory of 31 passive markers, including one marker placed specifically on the second sacral vertebrae, the closest anatomical point to the location of the CoM [27]. The remaining markers were placed across the lower limbs and the pelvis of the subjects based on the Helen Hayes protocol [28]. Figure 1b–d show the marker set used in both groups. The frequency rate of the MOCAP system and the force platforms were both set to 250 Hz. A total of ten trials per subject was recorded.

The datasets were analyzed using MATLAB (Mathworks, Inc., Natick, MA, USA) and the Visual 3D software (C-Motion, Inc., Rockville, MD, USA) was used for calculating the vertical CoM trajectory, joint trajectories, and the GRF, while the latter were also used to detect the gait events of heel-strike and toe-off.

### 2.3. CoM Spectral Analysis

The CoM parameter has an important role in the study of human motion. It has mainly been used as an estimator of human balance and posture. In a more dynamic scenario, the CoM trajectory reflects the interaction between different body segments to execute motion. For the case of walking, this type of gait can be defined as a series of up-down and medio-lateral motions of the CoM in the forward direction.

**Figure 2:**
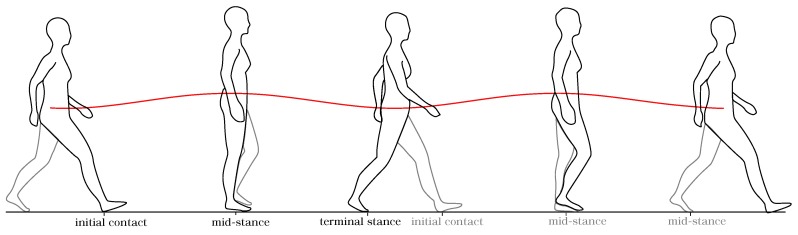
The body center of mass vertical trajectory during a gait cycle.

A typical vertical CoM displacement during a gait cycle is illustrated in Figure 2. It can be observed that the CoM trajectory describes a sinusoidal pattern in the vertical direction. The initial contact is taken as the reference point. From there, the CoM displaces upward as the body weight is loaded onto the trailing leg, reaching its highest value at midstance. After that, this value decreases until the leading leg meets the stance phase. The CoM repeats this pattern each new step. During a single gait cycle, the CoM vertical position has two minimal and maximal points, which correspond to the instants of initial contact and midstance of each leg. For the case of amputee gait, the vertical CoM trajectory is also a periodic signal, even though its pattern differs from the sine-wave shape found in the non-amputee case [20].

The periodic characteristic of the CoM trajectory regardless of the gait condition has encouraged research to use the discrete Fourier series (DFS) to mathematically represent the performance of this parameter during a gait cycle. For instance, in [19], the CoM trajectory was estimated from GRF measurements, which were expanded in terms of the DFS for each force component. The authors showed results from normal gaits in different experimental conditions and suggested the analysis of the DFS coefficients as an alternative method to characterize human gait. A more recent attempt is found in [29]. They analyzed consecutive strides at different velocities during walking and running. From these datasets, expressions of the DFS of the 3D CoM trajectory were obtained at each velocity condition. In [30], the CoM vertical trajectory is estimated from a *sum-of-sines* function whose input is the acceleration measured at the trunk segment.

According to the DFS formulation, a discrete periodic sequence $x\left[n\right]$ with a period of *N* samples can be represented as a sum of sinusoidal functions whose frequencies are multiple of the fundamental frequency $\omega_{0}=2\pi /N$. The DFS of $x\left[n\right]$ can be expressed as

(1) $$x\left[n\right]=\frac{1}{N}\sum_{k=1}^{N}a_{k}e^{jk(2\pi /N)n}.$$

Likewise, the DFS coefficients can be written as

(2) $$a_{k}=\sum_{k=1}^{N}x\left[n\right]e^{-jk(2\pi /N)n}.$$

As the sequence $x\left[n\right]$ has a period of *N* samples, its spectrum can only have components that are located in frequencies multiple of $\omega_{0}$. This means that the DFS coefficients will appear in frequencies defined as

(3) $$\omega_{k}=k\omega_{0},\;\;k=1,2,3,\ldots ,N.$$

To define how many DFS coefficients are necessary in order to achieve a good approximation of $x\left[n\right]$, the energy calculation of the signal is a useful criterion. The energy in the frequency domain can be calculated in terms of the DFS coefficients, as follows:

(4) $$E_{T}=\sum_{k=1}^{N}{\left|a_{k}\right|}^{2},$$

where ${\left|a_{k}\right|}^{2}$ is known as the average power of the *k*th component of the DFS.

Here, the Fourier analysis is used to represent the CoM vertical trajectory of each subject in terms of the DFS components. The CoM signals were collected from the a single marker placed on the sacral vertebrae.

### 2.4. DFS-Based Symmetry Index

As stated before, the vertical CoM trajectory also reflects the symmetry condition between the lower limbs, since the two oscillations of this parameter during a single gait cycle are the consequence of the two leg contacts with surface, which are expected to behave similarly in a symmetric gait.

For the case of walking at normal speed for a non-amputee subject, the CoM waveform behaves like an even function with respect to the vertical axis at a time equals to the step time, i.e., the fundamental period of this signal coincides to the step duration. In the context of the Fourier analysis, a symmetric pattern of the vertical CoM, expressed in DFS components, will contain only even coefficients.

In order to quantify the symmetry of the vertical CoM, an index that measures this condition can be thought in terms of the ratio between the energy from the even DFS components and the total signal energy. Considering that the even components make equal contribution to each vertical oscillation (one per step) of the CoM during a gait cycle, the presence of odd components will represent asymmetries on this signal. Based on this, an index of symmetry based on the DFS coefficients that synthesized the vertical CoM trajectory can be expressed as follows [29]:

(5) $$S_{CoM}=\frac{\sum_{i}{\left|a_{i}\right|}^{2}}{\sum_{k}{\left|a_{k}\right|}^{2}},\;\;i=2,4,6,\ldots ,N\;and\;k=1,2,3,\ldots ,N,$$

where $a_{i}$ are the even components, while $a_{k}$ are the overall DFS components for the studied waveform. A value of $S_{CoM}$ equal to 1 means perfect symmetry, i.e., the vertical CoM during the first step is equal to the one produced during the next step. In this work, the $S_{CoM}$ index is calculated to quantitatively measured the symmetry level of amputee gait compared to a non-amputee reference.

## 3. Results

**Figure 3:**
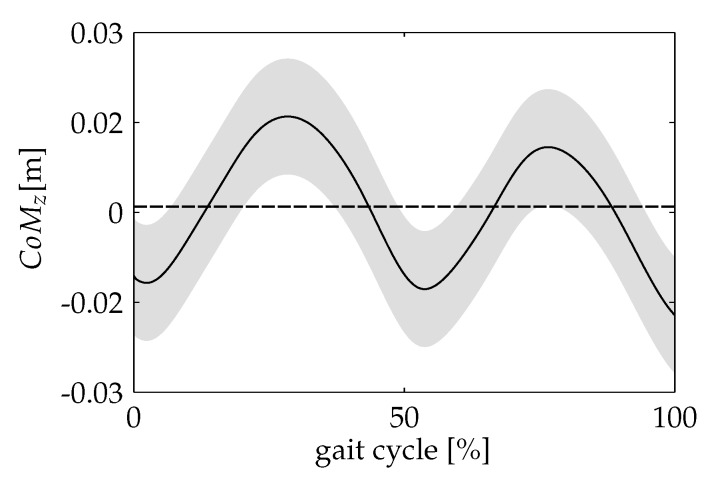
The CoM vertical excursion (mean ± SD) of the non-amputee group.

Figure 3 shows the CoM vertical trajectory for the non-amputee group. The trajectory corresponds to the mean curve of a single gait cycle for each trial of the entire control group, where the initial contacts (at the moment of the heel strike event) of the same limb were taken as the initial and the final point of the stride. The obtained pattern clearly shows a periodic signal with two periods corresponding to each step. The highest value occurs at midstance of the trailing leg, and it is repeated at the same instant in the contralateral limb. The lowest points coincide with the feet contacts at each new step.

**Figure 4:**
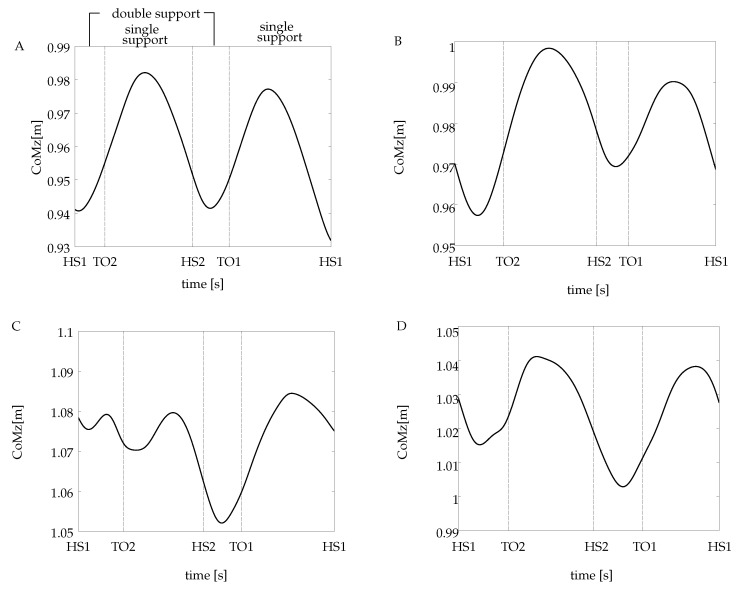
Vertical displacement of the CoM for (**A**) the non-amputee and (**B**–**D**) the amputees’ volunteers. $HS_{1}$ and $HS_{2}$ correspond to heel strike events of the trailing and the leading leg, respectively. The $TO_{1}$ and $TO_{2}$ are toe-off events for the same legs. For the case of the amputee subjects, the trailing leg corresponds to the intact leg.

Different patterns of the CoM trajectory were obtained for each amputee subject, all shown in Figure 4. To facilitate the comparison to the non-amputee case, a reference subject from this group has been selected, which corresponds to subject A (see Figure 4A). The abscissa is divided in *heel strike* and *toe-off* events, which delimit the single support and the double support periods of the gait cycle. The illustrated trajectories correspond to a single recorded trial for each subject. It is worth pointing out that all the calculations (the CoM spectral analysis and the DFS-based symmetry index) were carried out using all the waveforms extracted from the non-amputee chosen as a reference and all the subjects of the amputee group.

The CoM waveforms from all the amputees have some characteristics that differ from the non-amputee pattern. First, for the case of subjects B and D, the CoM displaces downwards from its initial position at first double support, different from the ascendant behavior in subject A. Second, during the final double support, when it takes place the step-to-step transition, the CoM height from the amputee group is not the same as the one at the initial contact. Instead of that, the vertical CoM goes to a lower position for the case of subjects C and D and an upper position for subject B.

On the other hand, as it is exhibited on the non-amputee reference curve, the CoM trajectory for subjects B and D raises after the end of the initial double support, attaining the highest point at midstance (the middle point at the initial single support). An interesting behavior was found in the CoM pattern from subject C, presented in Figure 4C. It exhibits two small oscillations around the initial position during the first step. These oscillations do not appear in the next step.

**Figure 5:**
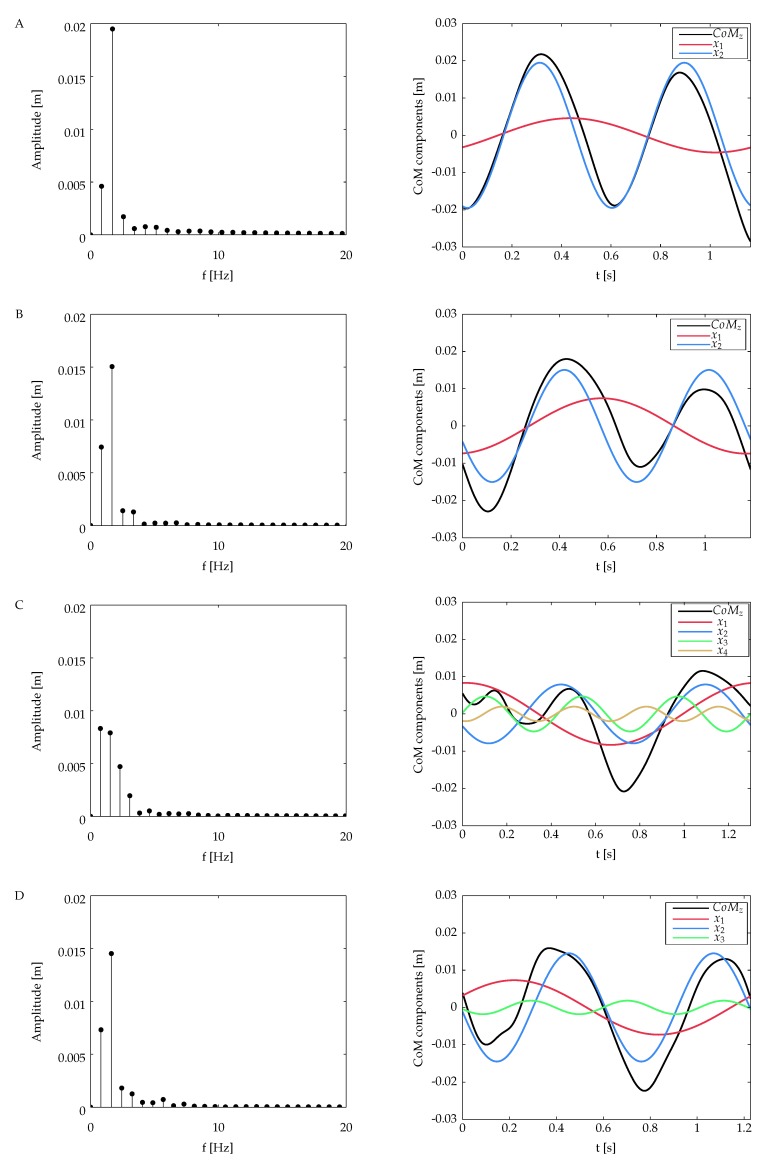
Magnitude spectrum (first column) and decomposition of the vertical CoM signal into its harmonics (second column) for (**A**) the non-amputee and (**B**–**D**) the amputees’ volunteers. The black curve corresponds to the original waveform.

The DFS magnitude spectrum of the vertical CoM for all the subjects is presented in Figure 5. The individual sinusoidal components in time domain are also presented for each case. The number of the DFS coefficients calculated for each vertical CoM is related to the energy criterion defined in Equation (4): the reconstruction of the CoM waveform includes the DFS coefficients that represent up to 99% of the total energy signal. Table 1 summarizes the number of DFS coefficients for each subject and their contribution to the overall signal energy.

The CoM harmonics obtained from subject A (see Figure 5, first row) present two oscillations with almost the same amplitude and duration in the course of the gait cycle. The magnitude spectrum of this subject reveals two main coefficients for k=1 and k=2. The first one constitutes approximately the 5% of the total energy signal, while the component in k=2 has 94% of the energy signal. The latter oscillates at a frequency very close to the inverse of the step period of this subject ($t_{step}=0.58$).

From the amputee group, subjects B and D have a similar behavior; the signal energy is concentrated in two DFS coefficients in k=1 and k=2, which have approximately 20% and 79% of the total signal energy, respectively, in frequencies that coincide with one time and twice the frequency of the gait cycle (see Table 1). A different behavior is observed in subject C; the energy signal is spread out into four coefficients, with the first two having almost the same contribution in terms of energy.

Table 2 shows the DFS-based symmetry index values for all the subjects, which was calculated using Equation (5). Subject A has the highest value. For the case of the amputee group, the $S_{CoM}$ values in decreasing order correspond to subjects B, D, and C.

## 4. Discussion

The symmetry analysis in walking has been a matter of discussion in many clinical papers, but still there are different points of view regarding how it can be accurately measured. For the case of the amputee gait, literature lacks of quantitative methods to assess gait symmetry. Given this scenario, this work examines the CoM vertical trajectory during walking using an approach based on the DFS.

In this work, a symmetry analysis of amputee gait is performed employing the CoM vertical trajectory. Here, the obtained results are discussed in view of the specific gait patterns exhibited by each subject and also another gait symmetry measure proposed in the literature, particularly the symmetry magnitude, $S_{M}$. This specific index, which was proposed in [17], corresponds to the cross-correlation coefficient between analogous signals taken from both limbs. Specifically, the $S_{M}$ index is intended to look for similarities between two signals, one time-shifted with respect to the other one. At a time when the two sequences best match, the cross-correlation coefficient will approach to one. Table 3 and Table 4 list the values of $S_{M}$ calculated for joint kinematics and kinetic information extracted from the two groups of subjects. For the case of the vertical CoM, the signals to be compared correspond to the steps performed by each limb during a gait cycle. These results may be directly compared to those results in Table 2, i.e., the values in both tables were computed from data acquired in the same trials.

In our experiments, the vertical CoM of subject A does not exhibit a perfectly symmetric pattern, which results in a DFS with two coefficients. The first coefficient with 5% of the total energy signal is associated with the small difference in amplitude between the CoM oscillations. A similar result was found in [19] from measurements of non-amputees. The authors called the first harmonic the *Fundamental Extrinsic Harmonic* (FEH), since it contributes to the asymmetric characteristics of the vertical CoM. On the other hand, the second harmonic belongs to the *Fundamental Intrinsic Harmonics* (FIH), which reflects the symmetry aspects of the signal. The symmetry behavior of the reference subject is also reflected in the results of the $S_{M}$ index values of the measured variables shown in Table 3 and Table 4. All the $S_{M}$ values are higher than 0.9, which according to [17], mean that the two corresponding compared signals present high similarity. The spectral analysis for the amputee group identify at least two coefficients for each DFS from every subject. For all the cases, including the non-amputee’s, the second component is related to the symmetrical pattern of the vertical CoM, which oscillates at a frequency very close to the step frequency. The rest of the components distorts the CoM signal in an amount proportional to their energy contribution.

For the cases of subjects B and D, two coefficients concentrate most of the energy signal. The fundamental component contributes with approximately 19% of the total energy, and it is related to the difference in amplitude and duration between steps. Even though in subject D the energy criterion includes a third coefficient, it only contributes with 1.22% of the total energy signal (see Table 1), so it can be simply discarded for the purpose of the present analysis. The value of the $S_{CoM}$ index for subjects B and D reflects the contribution of the fundamental odd coefficient in the reduction of the symmetry condition.

The CoM pattern from subject C is defined by four components, being the first one responsible for some of the vertical displacement of the resultant waveform, while the third and fourth components produce the initial oscillations, as well as the pronounced vertical excursion at the step-to-step transition. The sum of these individual components gives as a final result a very asymmetric pattern of the vertical CoM, with the lowest $S_{CoM}$ value of all the analyzed subjects.

Further analysis for the amputee group may be conducted taking into account the $S_{M}$ values. Higher $S_{M}$ values (see Table 3 and Table 4) are found in the hip and the knee angle in the sagittal plane, as well as in the vertical component of the GRF. As the $S_{M}$ index measures the difference in magnitude of two time sequences, that is, the sequences corresponding to the gait variables from each limb; high values do not necessarily mean that the amputee gait does not present any constraint that favors compensatory mechanisms. It means that each side of the subject has the same behavior, but can be different if compared to the same variable measured on the control subject. A careful inspection of the angles waveforms would probably reveal the presence of compensation mechanisms that would lead to different behaviors of the intact and the amputee limbs in contrast to a non-amputee gait.

In contrast, the lowest $S_{M}$ values are found in the hip frontal angle, the ankle sagittal angle, and the vertical CoM trajectory. These values are probably the result of a hip hiking mechanism and the absence of actuation of the ankle joint on the prosthetic leg.

Regarding the trajectory of the CoM, the $S_{M}$ index indicates a low level of symmetry in subjects B and C, whereas the trajectory of subject D apparently has a symmetric behavior very close to the reference. Looking at Figure 4, it can be seen that subjects B and D indeed present a similar CoM pattern, which differs from the one obtained from the reference subject. The DFS-based index correctly ranked both subjects, i.e., subjects B and D presenting similar gait asymmetry, and $S_{COM}^{B}>S_{COM}^{D}$.

When analyzing joint, GRF, and CoM trajectories, one may infer that subject C presented an increased level of gait asymmetry. Both indexes ranked the performance from subject C as the most asymmetric. The low values at the pelvis frontal angle (obliquity), at the hip frontal angle, and at the ankle sagittal angle may have impacted the the CoM symmetry between steps.

Despite the successful assessment of gait symmetry using the CoM trajectory, the proposed method and experimental protocol still present some limitations. First, the setup for the gait analysis experiment was defined to validate the proposed method without any “perturbation” that could alter the self-selected pattern of walking of subjects. In this manner, the results showed no significant variability between consecutive strides in the CoM trajectories of either the control group nor the amputee group. This fact excludes the phenomenon of inter-stride variability [31].

Furthermore, in the current study, the CoM trajectory was obtained using an optical motion capture system. However, possibility of assessing gait symmetry in real-life conditions depend on using an uncomplicated wearable sensor to estimate vertical CoM displacement. For instance, joint kinematics may be estimated using both inertial sensors [32] or optical fiber [33]. Instrumented insoles may also be used, including for controlling lower-limbs prosthesis [34]. The method to provide such estimate robustly is yet to be developed.

## 5. Conclusions

In a purely mechanical perspective, the trajectory of the CoM reflects the interaction between different body segments to execute motion. The dissimilarities between the vertical CoM in an amputee gait with respect to a non-amputee condition can be thought of as the manifestation of gait constraints and the developed compensatory mechanisms.

Specifically, the differences in the vertical excursion between steps may be related to unequal weight loading, which makes the subject to spend more time on the intact limb, increasing the weight born onto this leg. This kind of strategy is very common in the lower limb amputee population. Conversely, other compensatory mechanisms may have influenced the initial small oscillations of the vertical CoM pattern of one of the subjects.

The spectral-based analysis revealed the presence of several components multiple of the gait cycle frequency for the case of the amputee subjects. From the assumption of even symmetry of the CoM waveform in a normal situation, the presence of odd components was related to distortion of the resultant CoM represented by the selected components using an energy criterion. This relationship was established employing a symmetry index, $S_{CoM}$. As expected, the reference non-amputee subject exhibited the highest value, while the amputee group results reflected low levels of symmetry caused by the presence of odd DFS components with an important contribution concerning the overall energy signal.

The symmetry analysis performed using the $S_{M}$ index also revealed asymmetries in the amputee population. The results of the $S_{M}$ can be used to identify the most influential parameters to assess limb symmetry in a condition of unilateral transfemoral amputation. Different behaviors between limbs have a direct association to the asymmetric condition of the CoM trajectory.

The results presented here can be used to preliminary assess gait symmetry using only the vertical CoM trajectory. Indeed, this method could be applied for daily real-life walking, and not only walking under the supervision of the clinician inside a laboratory. Several recent studies have indicated that gait performance in a laboratory setting differs from real life [35,36,37]. In this sense, future works will focus on validating these results in a larger study involving non-amputees and amputees using an inertial sensor-based system on a daily basis. The study may also help with identifying specific attributes of gait asymmetry, which is an important property both for diagnostic and therapeutic interventions. In addition, feasibility studies may be conducted to investigate the ability to quantify gait asymmetry in different populations, such as stroke survivors featuring hemiparetic gait.

## Figures and Tables

**Table 1:** Coefficient frequencies and its energy contribution to the DFS of the vertical CoM.

Subject	Coefficient Frequency [Hz]	Coefficient Energy [%$E_{T}$]
A	$f_{1}=0.856$	$P_{1}=5.204$
	$f_{2}=1.712$	$P_{2}=93.359$
B	$f_{1}=0.839$	$P_{1}=19.328$
	$f_{2}=1.678$	$P_{2}=79.320$
C	$f_{1}=0.767$	$P_{1}=43.684$
	$f_{2}=1.534$	$P_{2}=39.450$
	$f_{3}=2.301$	$P_{3}=14.000$
	$f_{4}=3.067$	$P_{4}=2.409$
D	$f_{1}=0.812$	$P_{1}=19.753$
	$f_{2}=1.623$	$P_{2}=78.033$
	$f_{3}=2.435$	$P_{3}=1.220$

**Table 2:** DFS-based symmetry index values of the vertical CoM for all subjects.

Subject	$S_{\mathrm{CoM}}$
A	0.959
B	0.804
C	0.421
D	0.788

**Table 3:** $S_{M}$ index for the joint kinematics.

Subject	Pelvis	Hip		Knee Sagittal	Ankle Sagittal
		Sagittal	Frontal		
A	0.975	0.974	0.907	0.986	0.933
B	0.902	0.933	0.695	0.895	0.848
C	0.788	0.950	0.608	0.973	0.611
D	0.903	0.949	0.499	0.947	0.575

**Table 4:** $S_{M}$ index for the GRFs and the vertical CoM.

Subject	GRF		CoM
	Anterior-Posterior	Vertical	
A	0.998	0.978	0.956
B	0.956	0.955	0.556
C	0.821	0.954	0.501
D	0.744	0.834	0.950
